# RETRACTED: Ezz Eldin et al. Design and Synthesis of Novel 5-((3-(Trifluoromethyl)piperidin-1-yl)sulfonyl)indoline-2,3-dione Derivatives as Promising Antiviral Agents: In Vitro, In Silico, and Structure–Activity Relationship Studies. *Pharmaceuticals* 2023, *16*, 1247

**DOI:** 10.3390/ph18020226

**Published:** 2025-02-07

**Authors:** Rogy R. Ezz Eldin, Marwa A. Saleh, Sefat A. Alwarsh, Areej Rushdi, Azza Ali Althoqapy, Hoda S. El Saeed, Ayman Abo Elmaaty

**Affiliations:** 1 Pharmaceutical Organic Chemistry Department, Faculty of Pharmacy, Port Said University, Port Said 42526, Egypt; 2 Pharmaceutical Organic Chemistry Department, Faculty of Pharmacy (Girls), Al-Azhar University, Cairo 11651, Egypt; marwasaleh577@yahoo.com (M.A.S.); hodasamir3185@gmail.com (H.S.E.S.); 3 Department of Science, Prince Sultan Military College of Health Sciences, Dhahran 31932, Saudi Arabia; salwarsh@psmchs.edu.sa; 4 Department of Medical Microbiology and Immunology, Faculty of Medicine for Girls, Al-Azhar University, Cairo 11651, Egypt; areejrushdi@azhar.edu.eg (A.R.); azzaaly.micro@azhar.edu.eg (A.A.A.); 5 Medicinal Chemistry Department, Faculty of Pharmacy, Port Said University, Port Said 42526, Egypt

The *Pharmaceuticals* Editorial Office retracts the article “Design and Synthesis of Novel 5-((3-(Trifluoromethyl)piperidin-1-yl)sulfonyl)indoline-2,3-dione Derivatives as Promising Antiviral Agents: In Vitro, In Silico, and Structure–Activity Relationship Studies” [1] cited above.

Following publication, concerns were raised by the publisher regarding the validity of the data published in this article [1].

Adhering to our complaints procedure, an investigation was conducted by the Editorial Office and Editorial Board, which included a review of the raw data presented within this publication. While the authors fully cooperated with the investigation, they were unable to satisfactorily address a number of significant errors and anomalies found in the NMR and IR data. As a result, the Editorial Office and Editorial Board were unable to confirm the reliability of the findings and subsequently decided to retract the paper as per MDPI’s retraction policy (https://www.mdpi.com/ethics#_bookmark30, accessed on 25 October 2024) and in line with the Committee on Publication Ethics retraction guidelines (https://publicationethics.org/retraction-guidelines, accessed on 25 October 2024).

This retraction was approved by the Editor-in-Chief of the journal *Pharmaceuticals*.

The authors did not agree to this retraction.
